# Nosocomial Myiasis Caused by *Lucilia sericata* (Diptera: Calliphoridae) and Neonatal Myiasis by *Sarcophaga* spp. (Diptera: Sarcophagidae) in Mexico

**DOI:** 10.1155/2018/5067569

**Published:** 2018-10-02

**Authors:** Hugo Martínez-Rojano, Julio C. Noguez, Herón Huerta

**Affiliations:** ^1^Departamento de Posgrado e Investigación, Escuela Superior de Medicina del Instituto Politécnico Nacional, Plan de San Luis y Díaz Mirón s/n, Colonia Casco de Santo Tomas, Delegación Miguel Hidalgo C.P. 11340, Ciudad de México, Mexico; ^2^Laboratorio Estatal de Salud Pública del Estado de Hidalgo, Boulevard Luis Donaldo Colosio s/n, Col. Parque de Poblamiento, 42088 Pachuca, Hidalgo, Mexico; ^3^Laboratorio de Entomología, Instituto de Diagnóstico y Referencia Epidemiológicos, InDRE. Francisco de P. Miranda No. 177, Colonia Unidad Lomas de Plateros, 01480 Ciudad de México, Mexico

## Abstract

The presence of nosocomial myiasis reflects a lack of adequate medical attention, due to the physical facilities and/or the health care personnel. Patients requiring special attention are more susceptible, such as those with a loss of consciousness, assisted mechanical ventilation, tracheal tubes, or nasogastric probes. Nosocomial myiasis is a rare event that has a greater occurrence in the hospitals of poor and developing countries. The two cases herein described represent the first report of nosocomial myiasis in Mexico. The causal agents were found to be *Lucilia sericata* and *Sarcophaga* spp. The taxonomical identification of the larvae of the second and third instar was based on the morphology of the cephaloskeleton, anterior spiracles, and peritreme plaques.

## 1. Introduction

Nosocomial myiasis, first reported in 1980 [1], refers to an infestation originating in a hospital environment. The conditions for this form of infestation, unusual in the developed world, are more commonly found in poor and developing countries. It is likely that nosocomial myiasis is underreported in the latter countries and thus may be more frequent than commonly thought. Its presence reflects a lack of adequate medical attention due to the physical facilities and/or the health care personnel. Patients requiring special attention are particularly susceptible, such as those with a loss of consciousness, a stab wound, a draining abscess, assisted mechanical ventilation, a tracheal tube, a nasogastric probe, or any type of lesion resulting in the continuous presence of mucous and secretions [2].

Comatose and disabled patients are very much prone to suffering an infestation. In addition to the myiasis found in wounds [3], there are descriptions of nasal and nasopharyngeal [4, 5], oral-tracheal [6], ocular [7], urogenital [8], and intestinal [9] myiasis. Upon discovering nosocomial myiasis, the identification of the causative species is crucial in order to be able to recognize the habits of the diptera species in question and adequately plan its eradication. The following species have been recognized as causal agents of nosocomial myiasis: *Lucilia sericata* [10], *Megaselia scalaris* [11], *Sarcophaga* spp. [12], *Cochliomyia hominivorax* [13], *Cochliomyia macellaria* [14], and *Musca domestica* [15].

Nosocomial myiasis should alert medical personnel about the possible presence of other infestations. For example, one hospital had an infestation of mice, which attracted the flies that produced the cases of nasal myiasis in patients of the intensive care unit (ICU) [16]. Nosocomial myiasis reflects the quality of the medical staff responsible for the patients. Regarding hospital administration, simple measures that can help to prevent nosocomial myiasis are the placement of window screens and ventilation duct filters [17]. Another factor to be checked is the storage and handling of the food for patients [17].

The present report describes two cases of nosocomial myiasis, one in a patient of the ICU of a general hospital in Hidalgo State, Mexico, and the other of neonatal myiasis in a newborn that was admitted through the emergency service to the neonatal ICU in a hospital for maternity and infant care in Guerrero State, Mexico.

## 2. Case Presentation

We illustrate the temporal sequence of the salient points of the illness of each case by a timeline followed by a detailed description.

### 2.1. Case 1

A 57-year-old male patient, born and living in Hidalgo State, had suffered from hepatocellular carcinoma and underwent tumor surgery in June of 2017, followed by chemotherapy. He had been diabetic for 3 years, treated with metformin and glibenclamide, and showed poor control of blood glucose. He entered the general hospital of the Mezquital Ixmiquilpan Valley on April 25, 2018, with the diagnosis of acute abdomen and hypovolemic shock. This condition required resuscitation therapy and restitution of blood volume with crystalloid fluids. On the same day, an exploratory laparotomy was executed, finding hemoperitoneum of 2,000 mL caused by bleeding of the right lobe of the liver. Moreover, multiple adherences were observed of the omentum to the liver lobe, prompting the decision to perform perihepatic packing with plastic nonporous membrane. Another laparotomy had been programmed to take place in 24 hours. The patient was placed in the ICU and controlled with mechanical ventilation and inotropic and vasoactive drugs. The perihepatic packing was removed after 24 hours, revealing the persistence of bleeding. Consequently, the liver was again packed. On the first of May 2018, 30 whitish larvae with an average length of 1.1 cm were discovered in both nostrils. They were collected and sent to the entomology lab of the Institute of Diagnosis and Epidemiological Reference (InDRE, Instituto de Diagnóstico y Referencia Epidemiológicos) for taxonomical analysis. On May 2, the perihepatic packing was removed without complications, but the patient was in the grave condition with a poor short-term prognosis as a result of multiple organ dysfunction, which was the cause of death on May 15.

**Figure 1:**
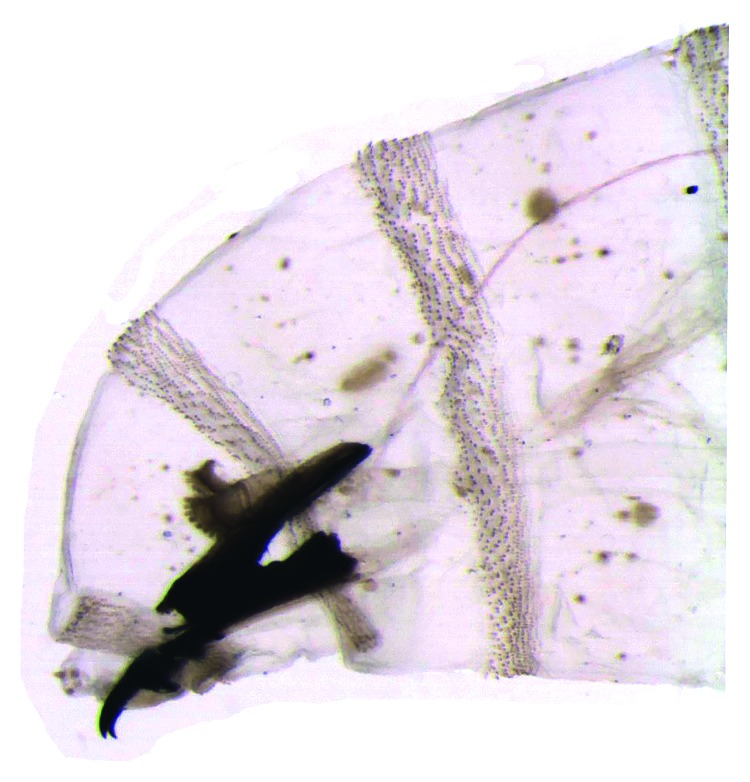
*Lucilia sericata* larva, third stage. Part of the anterior, cephaloskeleton and anterior spiracles.

**Figure 2:**
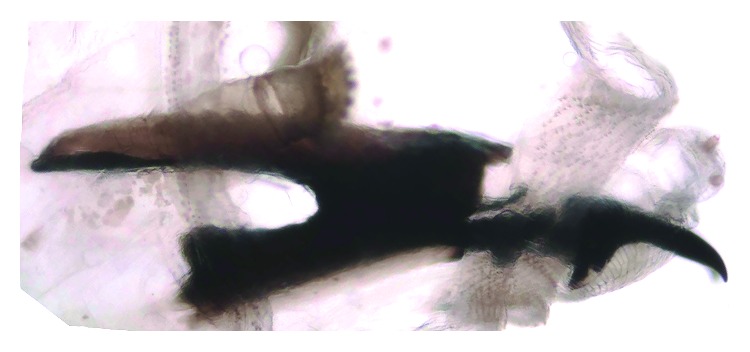
*Lucilia sericata*, Larva, third stage. Cephaloskeleton and anterior spiracle.

**Figure 3:**
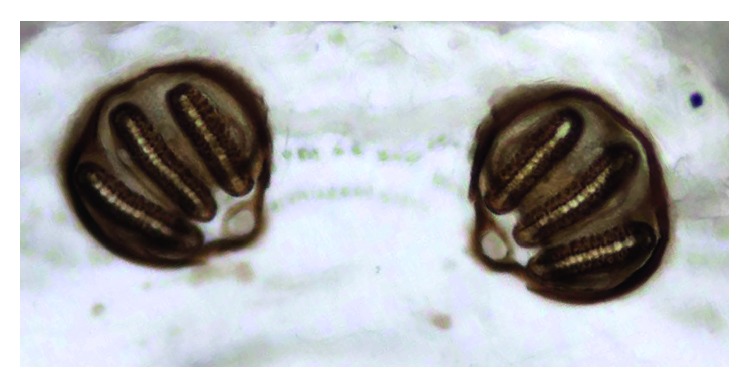
*Lucilia sericata* larva, third stage. Peritrema, posterior spiracles.

The taxonomical classification of the larvae of the third instar was based on the morphology of the cephaloskeleton, anterior spiracles (Figures 1 and 2), and peritreme plaques (Figure 3). These organisms were identified as *Lucilia sericata* (Diptera: Calliphoridae).

### 2.2. Case 2

**Figure 4:**
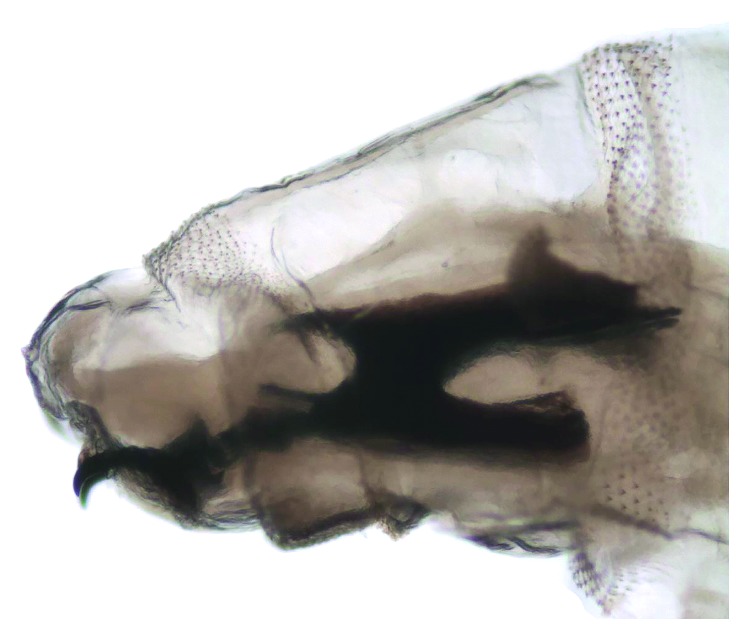
*Sarcophaga* spp. Larva, second stage. Part of the anterior, cephaloskeleton and anterior spiracle.

**Figure 5:**
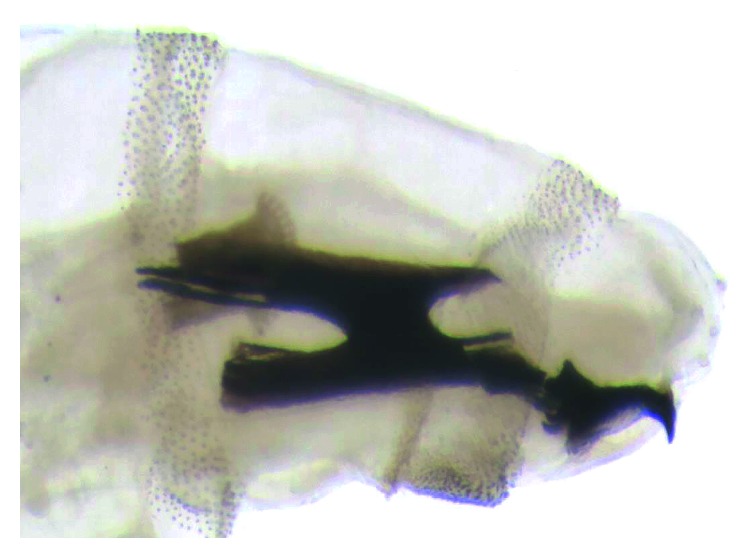
*Sarcophaga* spp. Larva, second stage. Cephaloskeleton and previous anterior spiracles.

**Figure 6:**
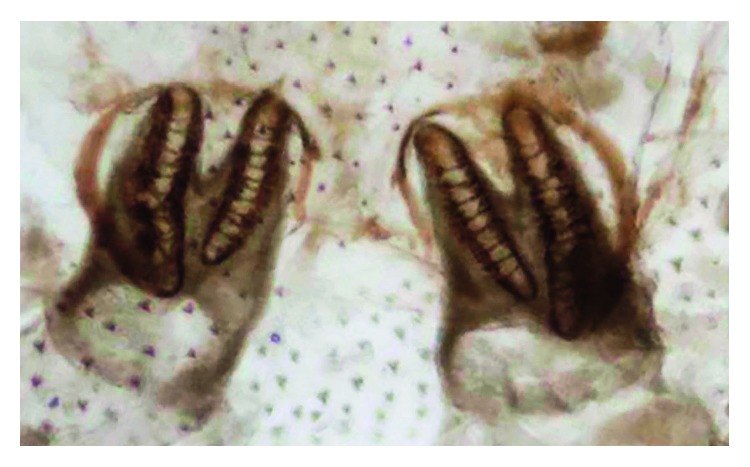
*Sarcophaga* spp. Larva, second instar. Peritrema, posterior spiracles with two slits.

A 24-day-old baby girl from Guerrero State was the product of the second gestation of an apparently healthy mother who underwent a normal pregnancy carried to full-term with vaginal delivery of a single product. The weight, height, and Apgar score of the newborn are unknown. The newborn was nursed by the mother for the first 7 days of life. At 10 days of age, the infant received an insect bite, which led to fever after 48 hours. The parents took the baby to the health center, where an infection of the respiratory tract and hyporexia were detected. Upon arrival, the baby was found in generally poor condition, weighing 2,220 g and suffering from severe dehydration. Intravenous feeding was begun immediately to stabilize the infant's condition, and subsequently, she was transferred to the Hospital of Indigenous Mothers and Children of Guerrero. At that facility, the baby arrived in a state of cardiac arrest, prompting cardiopulmonary resuscitation and phase III ventilatory support. Antibiotic treatment was given, and hydrotherapy was continued due to the presence of sepsis and septic shock. Two days posthospitalization, the presence of fly larvae was observed in both nostrils, giving rise to ivermectin treatment. Four whitish larvae were extracted, having an average length of 1.2 cm. They were sent to the Entomology Lab of the InDRE for taxonomical classification. The identification of the taxonomy of the larvae of second instar was based on the morphology of the cephaloskeleton, anterior spiracles (Figures 4 and 5), and peritreme plaques (Figure 6). They remained an undefined *Sarcophaga* sp. (Diptera: Sarcophagidae), as the morphology did not correspond to any common or uncommon species known to cause myiasis. Diverse cases have been reported of traumatic and intestinal myiasis provoked by *Sarcophaga* sp., but the specific species has not been determined [18].

## 3. Discussion

To investigate the situation of nosocomial myiasis in Mexico, a search of the following databases was carried out in Spanish and English: Literatura Latinoamericana y del Caribe en Ciencias de la Salud (LILACS: http://lilacs.bvsalud.org), Scientific Electronic Library Online (SciELO: http://www.scielo.org), PubMed (http://www.ncbi.nlm.nih.gov/pubmed), EBSCOhost (http://www.ebscohost.com), and Google Scholar (http://scholar.google.com). Since no publications were found for nosocomial or neonatal myiasis in Mexico, the cases herein described represent first report.

By definition, a nosocomial myiasis has its origin in a hospital stay [19]. Generally, a myiasis is considered nosocomial when the manifestation appears three days or more after admission to the hospital [20]. In case 1 of the present report, larvae were discovered 6 days postadmission to the ICU. In case 2, they were observed on the fourth day of the hospital stay. The causal agents linked to nosocomial myiases belong to the genera *Sarcophaga*, *Oestrus*, *Gasterophilus*, *Cochliomyia*, *Lucilia*, *Chrysomya,* and *Musca* [4, 11, 17, 19]. Myiasis has also been reported involving *Dermatobia hominis* (Diptera: Cuterebridae) [13].

*Lucilia sericata* is an ectoparasite commonly known as the green bottle fly. Open wounds with necrotic areas are ideal sites for the deposition of eggs and the development of larvae. Under such conditions, it takes about 2.5 days from the egg laying to the first stage of larvae [11, 21]. Hence, the two cases included herein constitute examples of nosocomial myiasis.

In these patients, the alteration in the state of consciousness and the hypoesthesia could have impeded the patient from detecting the presence of the fly [17]. This coincides with the reports of myiasis reported in hospitals in other parts of the world. The majority of patients with myiasis are debilitated by a concomitant condition, such as diabetes, a vascular disorder, advanced cancer, open wounds, hemorrhaging that is not visible around the site of a tracheotomy, halitosis with an underlying pulmonary bacterial infection, or any extremely grave condition [17, 19].

According to clinical cases reported in the Middle East and America, a common condition is assisted mechanical ventilation or a nasogastric probe [10], which coincides with the condition of the two patients currently reported. The ventilation apparatus facilitates the presence of secretions and saliva, attractive substrates for oviposition. Consequently, the most common site to find the larvae of nosocomial myiasis is the naval cavity [22].

Additionally, nosocomial myiases reported in various parts of the world are associated with the ICU. However, in Latin American and the Caribbean, the documented cases were not infested in this hospital area. The infestation of the present case 1 seems to have occurred in the ICU, probably due to the ground floor location of the same, which leaves it exposed to conditions that could lead to the proliferation of flies and infestation [23]. The myiasis of case 2 most likely had its origin in the maternal-infant hospital's emergency service [17], a unit typically exposed to the movement of patients and their family, making it a relatively uncontrolled area. This situation lends itself to the entry of flies and their contact with patients [17].

Although nosocomial myiasis is uncommon, there are reports from Iran, Korea, Taiwan, Canada, Costa Rica, Jamaica, Honduras, French Guiana, and Brazil. The risk factors in these cases include secretions, blood and mucous around a wound, an odor of decomposition, inadequate medical care, limited infrastructure in the hospital environment, and the season of the year (spring-summer are the peak seasons for flies) [7–12]. On the other hand, it is generally considered that awareness of the full extent of the problem of nosocomial myiasis is restricted by underreporting, the result of certain hospital policies, the risk of lawsuits for malpractice, the lack of consciousness among the medical staff about the relevance of such reports, the lack of sufficient training of technical personnel, the difficulty of diagnosing the condition, and certain social and cultural situations [23].

The measures of prevention against nosocomial myiasis should be directly related to the particular species of the fly involved. The elimination of infectious and contagious waste, as well as efficient sanitation and hygiene measures complemented by some kind of insecticide, should minimize the density of flies. Moreover, sealed windows provide physical barriers, while bandaging and care of wounds, and attention to the hygiene of the patients can diminish the risk of attracting of the causal agent.

## Data Availability

Data will be freely available through the corresponding author upon request.
